# Proteolytic dysregulation in the skin: insight from rare monogenic skin diseases

**DOI:** 10.1007/s00441-026-04052-x

**Published:** 2026-03-04

**Authors:** Zhongtao Li, Sheng Wang, Diana C. Blaydon, David P. Kelsell

**Affiliations:** 1 Department of Dermatology, West China Hospital, Sichuan University, Chengdu, 610041 China; 2 Centre for Cell Biology and Cutaneous Research, Faculty of Medicine and Dentistry, Blizard Institute, Queen Mary University of London, Whitechapel, London, E1 2AT UK; 3 Laboratory of Dermatology, Clinical Institute of Inflammation and Immunology (CIII), Frontiers Science Center for Disease-Related Molecular Network, West China Hospital, Sichuan University, Chengdu, 610041 China

**Keywords:** Skin barrier, Keratinocyte, Desquamation, Serpin, Cathepsin

## Abstract

Proteases are essential enzymes that, through the breakdown of proteins, regulate many aspects of tissue homeostasis including barrier function, cellular signaling, and tissue repair mechanisms in organisms. Disease gene discovery in a number of monogenic skin diseases has deepened the knowledge of how proteases and protease inhibitors can regulate skin homeostasis, keratinocyte desmosome-mediated cell adhesion, and epidermal barrier function. This short review details the association of protease dysregulation with monogenic skin diseases, postulated disease mechanisms, and emerging therapeutic strategies.

## Introduction

The skin is the largest organ of the human body and is constantly exposed to the external environment. The outer epidermal layer plays a vital role in forming the protective skin barrier and is, in part, reliant on the integrated structure and function of intercellular adhesion and cell communication hubs; these are the desmosomes, gap, adherens, and tight junctions (Cohen-Barak et al. 2020; Jensen and Proksch 2009). However, the epidermis constantly undergoes a process of renewal whereby adhesions between the outermost, dead keratinocytes (corneocytes) need to be cleaved in a controlled manner to allow corneocyte shedding from the stratum corneum (termed desquamation). This desquamation process is a highly regulated process that requires a careful balance of proteases and their respective inhibitors (Stamatas 2024; Cohen-Barak et al. 2022).

**Fig. 1:**
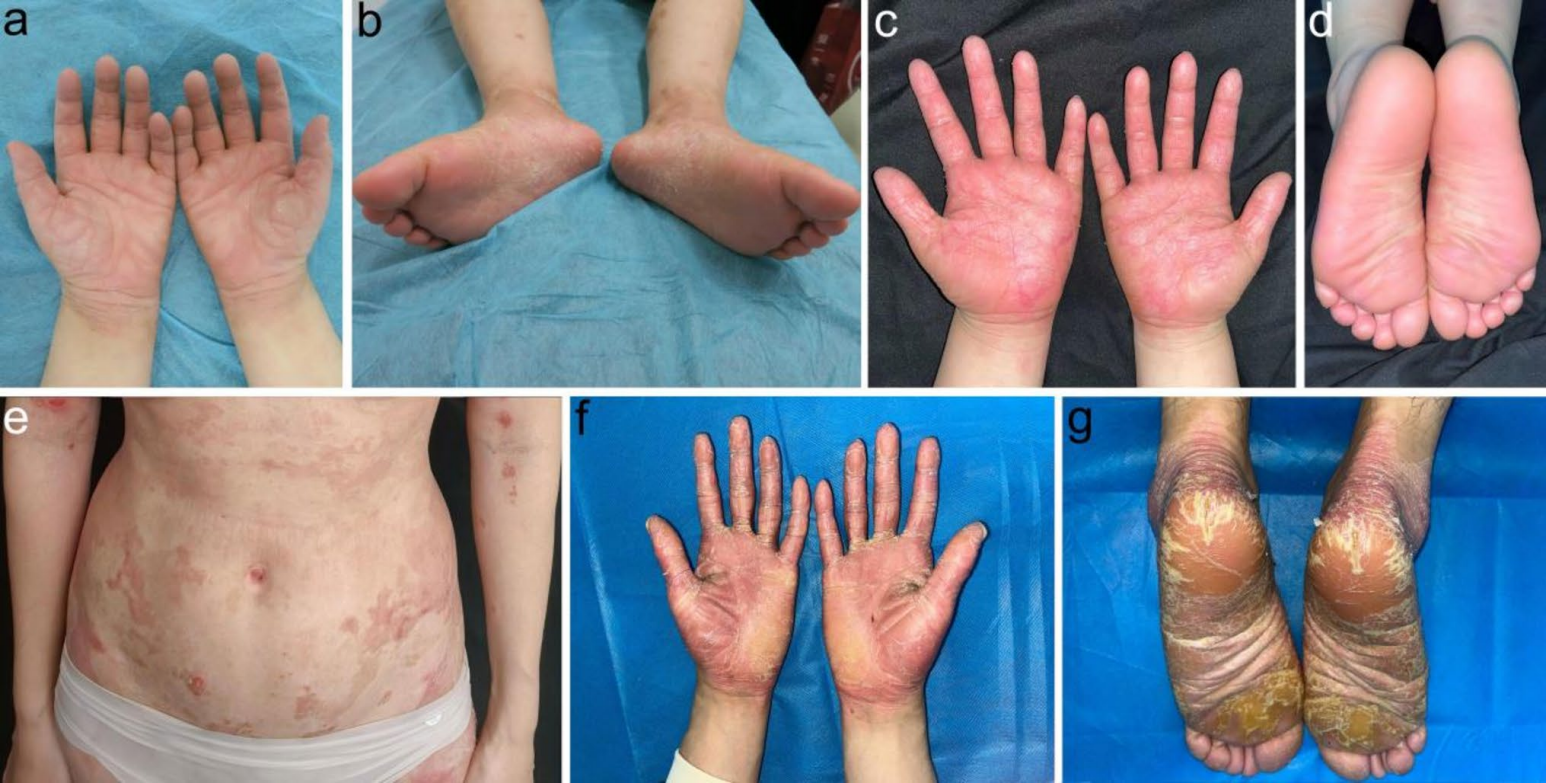
Clinical manifestations of hereditary skin diseases caused by pathogenic variants in genes encoding proteases or protease inhibitors. **a**, **b** Mild erythema and hyperkeratotic desquamation on palm and plantar surfaces, extending to the wrists and ankles in a 10-year-old boy carrying a homozygous pathogenic variant for *SERPINB7* c.796C > T. **c**, **d** Diffuse erythematous hyperkeratosis of the palms and plantar surfaces, extending to the wrists in an 8-year-old girl carrying a homozygous pathogenic variant for *SERPINA12* c.635-7A > G. **e** Extensive erythematous plaques with double-edged marginal scales on the trunk and extremities in a 23-year-old female carrying compound heterozygous pathogenic variants in *SPINK5* c.1048C > T and c.−97_−80del. **f**, **g** Erythema and hyperkeratosis on the palms and plantar surfaces in a 21-year-old male caused by compound heterozygous pathogenic variants in *CTSC* c.800T > C and c.1234T > A

Proteases are enzymes that catalyze the hydrolysis of peptide bonds in proteins and polypeptides. More than 500 proteases have been identified in the human genome (Donzelli et al. 2023) and are categorized according to their catalytic mechanisms, into serine, cysteine, aspartic, threonine proteases, and metalloproteases, though some categories contain families with two or more catalytic types (Fan et al. 2017). Among them, over 100 proteases are expressed in human skin with emerging different biological functions including epidermal barrier homeostasis, maintaining keratinocyte adhesion and regulation of cell signaling including those related to inflammation (Stewart-McGuinness et al. 2022). Many of these biological functions within the skin have been revealed from genetic and cell biology studies of rare monogenic disorders of epidermal keratinization (Table 1) (Has 2018). These include palmoplantar keratoderma (PPK; hyperkeratosis/callus formation on the palm and sole epidermis) and peeling skin syndrome (PSS; epidermal peeling of the palm and sole epidermis) (Fig. 1). These epidermal differentiation disorders (EDD) represent inherited disorders of keratinization characterized by abnormal epidermal differentiation, encompassing those that largely affect the palmoplantar (palm and sole skin, pEDD) and those that associate with nonsyndromic (nEDD) or syndromic skin conditions (sEDD) (Sprecher et al. 2025; Akiyama et al. 2025; Paller et al. 2025). This new nomenclature includes the causative gene associated with each specific EDD condition.

**Table 1:** Protease and protease inhibitors in hereditary skin diseases

Disease	Gene-based name	Clinical manifestation	MIM	Inheritance	Protease	Protease inhibitor	Inactive protease	Reference
Nagashima-type PPK	*SERPINB7*-pEDD	Mild diffuse hyperkeratosis with erythema, skin peeling, aquagenic whitening, palmoplantar hyperhidrosis on the palms and soles accompanied with odor	615598	AR	Legumain	SERPINB7	/	Kubo et al. 2013
*SERPINA12*-related PPK	*SERPINA12*-pEDD	Diffuse hyperkeratosis with erythema, skin peeling, aquagenic whitening, palmoplantar hyperhidrosis on the palms and soles accompanied with odor	/	AR	KLK7	Vaspin	/	Mohamad et al. 2020
Exfoliative ichthyosis/PSS5	*SERPINB8*-pEDD	Skin peeling, hyperkeratotic plaques, and erythema on the palms and soles	617115	AR	Furin	SERPINB8	/	Pigors et al. 2016
Netherton syndrome	*SPINK5*-sEDD	Skin desquamation, atopic manifestation, hair abnormality	256500	AR	KLK5 KLK7 KLK14	LEKTI	/	Chavanas et al. 2000
Ichthyosis-hypotrichosis syndrome	*ST14*-sEDD	Diffuse scaling and hypotrichosis on the body, photophobia	602400	AR	Matriptase	/	/	Alef et al. 2009
Papillon-Lefèvre syndrome	*CTSC*-pEDD	Symmerical diffuse PPK, early loss of teeth, destructive periodontitis, and recurrent pyogenic infections	245000	AR	Cathepsin C	/	/	Toomes et al. 1999; Ghanei et al. 2021
Haim-Munk syndrome	*CTSC*-pEDD-arachnodactyly	Red, scaly, thickened skin on the palms and soles, alongside pus-producing skin infections, periodontitis, pes planus, onychogryphosis, arachnodactyly and acroosteolysis	245010			/	/	Hart et al. 2000; Chitsamankhun et al. 2024
Keratolytic winter erythema	*CTSB*-pEDD	Recurrent palmoplantar erythema and epidermal peeling, accompanied with itching, hyperhidrosis and odor, often worsen in winter	148370	AD	Cathepsin B	/	/	Hull et al. 2013; Ngcungcu et al. 2017
Acral peeling skin syndrome with exfoliative ichthyosis/PSS4	*CSTA*-nEDD	Dry, scaly skin over most of the body with coarse peeling of nonerythematous skin on the palms and soles, which is exacerbated by excessive moisture and minor trauma	607936	AR	Cathepsin L Cathepsin V	Cystatin A	/	Blaydon et al. 2011a
Ectodermal dysplasia 15, hypohidrotic/hair type	*CST6*-sEDD	Hypotrichosis, dry skin, eczema, blepharitis, photophobia and impaired sweating	618535	AR	Cathepsin L Cathepsin V Legumain	Cystatin M/E	/	van den Bogaard et al. 2019
Keratosis follicularis spinulosa decalvans	*CST6*-sEDD-cicatricial	Generalised follicular hyperkeratosis, dry skin, progressive cicatricial alopecia mainly on the scalp, facial erythema, folliculitis, and eye symptoms	612843	AD			/	Eckl et al. 2021
PLACK syndrome	*CAST*-pEDD	Peeling skin, leukonychia, acral punctate keratosis, cheilitis, and knuckle pads	616295	AR	Calpain	Calpastatin	/	Lin et al. 2015
Inflammatory skin and bowel syndrome	*ADAM17*-sEDD	Perioral and perianal erythema with fissuring and a generalized pustular rash that developed into psoriasiform erythroderma, with flares of erythema, scaling, and widespread pustules, associated with bowel disease	614328	AR	ADAM17	/	/	Blaydon et al. 2011b
Tylosis with oesophageal cancer	*RHBDF2*-pEDD	PPK, oral and oesophageal leukoplakia, and a significantly high lifetime risk of oesophageal squamous cell carcinoma	148500	AD	ADAM17	/	iRHOM2	Blaydon et al. 2012
Olmsted syndrome	*MBTPS2*-pEDD	Bilateral mutilating transgredient PPK and periorificial keratotic plaques with considerable clinical heterogeneity	300918	XLR	S2P	/	/	Haghighi et al. 2013
IFAP syndrome with or without BRESHECK syndrome	*MBTPS2*-sEDD-IFAP	Ichthyosis follicularis with atrichia and photophobia with or without additional features (e.g., corneal opacifications, developmental delay, skeletal malformations, ectodermal dysplasia, renal anomalies)	308205			/	/	Oeffner et al. 2009; Naiki et al. 2012
Keratosis follicularis spinulosa decalvans	*MBTPS2*-sEDD-cicatricial alopecia	Widespread hyperkeratotic follicular papules, facial erythema, hypotrichosis and scarring alopecia	308800			/	/	Aten et al. 2010

*PPK* palmoplantar keratoderma, *pEDD* palmoplantar epidermal differentiation disorder, *nEDD* nonsyndromic epidermal differentiation disorder, *sEDD* syndromic epidermal differentiation disorder, *PSS* peeling skin syndrome, *AR* autosomal recessive, *AD* autosomal dominant, *XLR* X-linked recessive

## Serine proteases and their inhibitors (serpins)

**Fig. 2:**
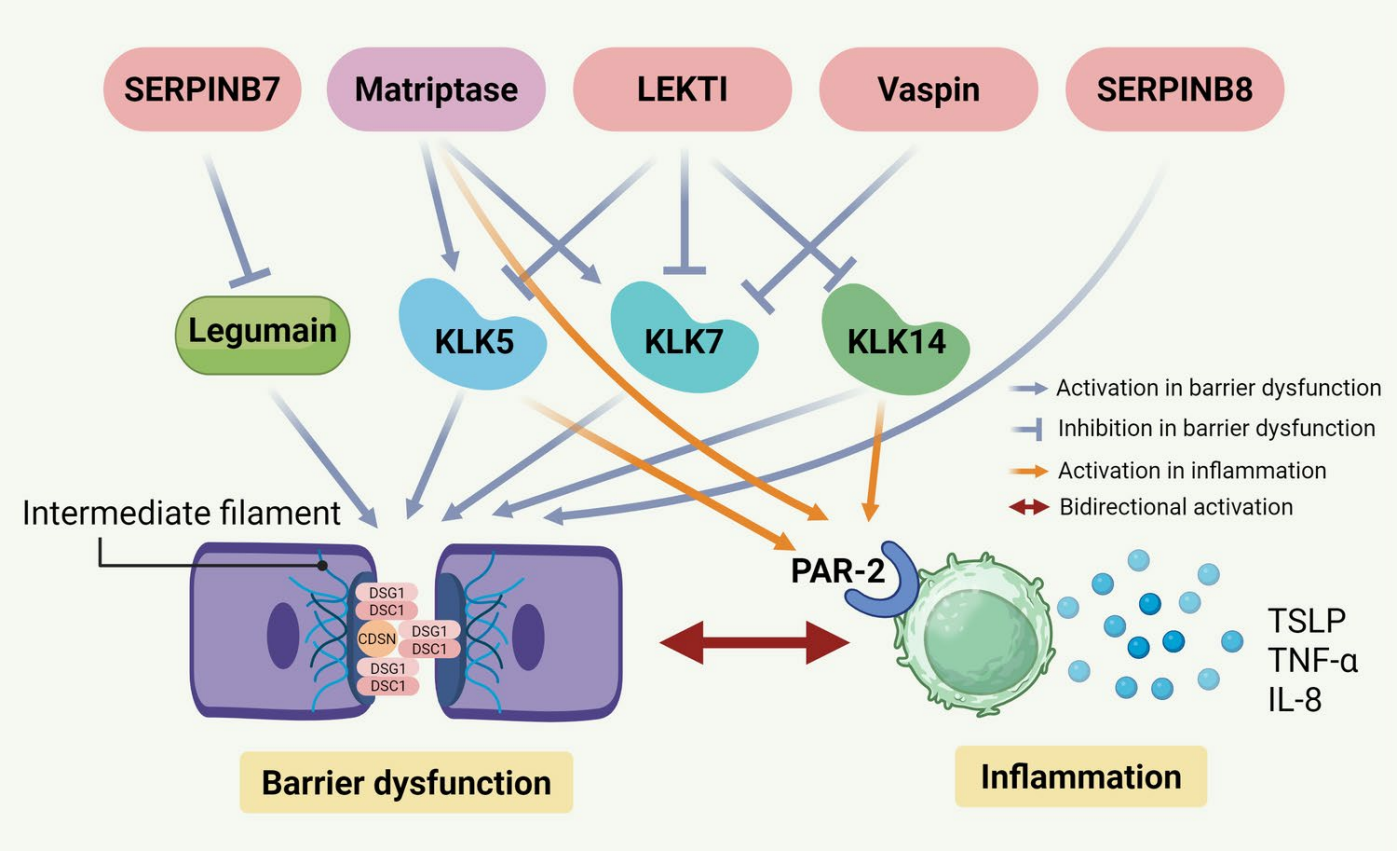
Schematic representation of serine protease and serpin regulation in skin barrier function and inflammation (created in https://BioRender.com). Serine proteases and serpins contribute to barrier dysfunction by degrading corneodesmosome components (DSG1, DSC1, and CDSN) and intermediate filaments (blue activation or inhibition arrows). They also activate PAR-2 on immune cells, inducing the release of cytokines such as TSLP, TNF-α, and IL-8, which result in cutaneous inflammation (orange activation arrows). Skin barrier dysfunction and inflammation reciprocally amplify each other, forming a self-reinforcing cycle (red activation arrow). LEKTI, lympho-epithelial Kazal-type related inhibitor; KLK, kallikrein-related peptidase; DSG1, desmoglein 1; DSC1, desmocollin 1; CDSN, corneodesmosin; PAR-2, protease-activated receptor 2; TSLP, thymic stromal lymphopoietin

Serine proteases are present in all living cells (Sil et al. 2024). Kallikrein-related peptidases (KLKs) are the largest family of trypsin- or chymotrypsin-like secreted serine proteases in humans (Meyer-Hoffert 2009). Among them, KLK5, KLK7, and KLK14 are reported to be the most important in desquamation (Ulbricht et al. 2018) and are involved in the cleavage of corneodesmosomes, the main inter-cellular adhesion junctions between corneocytes formed by desmoglein 1 (DSG1), desmocollin 1 (DSC1), and corneodesmosin (CDSN). KLK activity can also regulate the protease-activated receptor 2 (PAR-2) pathway, which in turn modulates pro-inflammatory signals, including thymic stromal lymphopoietin (TSLP), IL-8 and TNF-α (Zani et al. 2022). Overactive KLKs consequently impair the skin barrier and increase inflammation (Pontone et al. 2022). Importantly, epidermal barrier dysfunction and skin inflammation form a self-reinforcing pathogenic loop in inflammatory skin diseases (Beck et al. 2002) (Fig. 2). Serpins (serine protease inhibitors) are the largest family of all the protease inhibitors and use conformational changes to inhibit proteases. Though most serpins target serine proteases, some have been linked to inhibit papain-like cysteine proteases or caspases (Irving et al. 2002; Ray et al. 1992). To date, five monogenic autosomal recessive skin disorders are associated with dysregulated serine protease activity (Table 1).

Nagashima-type palmoplantar keratoderma [NPPK (*SERPINB7*-pEDD), MIM #615598] is associated with biallelic loss of function (LOF) variants of *SERPINB7* (Kubo et al. 2013; Kubo 2025), and *SERPINA12*-related palmoplantar keratoderma [SPPK (*SERPINA12*-pEDD)] is linked to biallelic LOF variants in *SERPINA12* (Mohamad et al. 2020). *SERPINB7*-pEDD and *SERPINA12*-pEDD exhibit overlapping clinical features, including hyperkeratosis with transient erythema (skin redness) extending beyond the palmoplantar margins (Fig. 1a–d). Additional features include palmoplantar skin peeling, aquagenic whitening (upon exposure to water), increased sweating (hyperhidrosis), odor, and prone to fungal infection (Liu et al. 2023; Mohamad et al. 2020). Digenic inheritance of LOF variants in *SERPINB7* and *SERPINA12* have recently been associated with NPPK in China and Japan suggesting shared biology exists between these two serine protease inhibitors (Liu et al. 2024; Jiang et al. 2025; Ebata et al. 2025; Zhang et al. 2025). Further studies are needed to explore the interplay between SERPINB7 and SERPINA12 in the pathogenesis of NPPK phenotypes.

While cases have also been reported in non-Asian populations (Hannula-Jouppi et al. 2020), *SERPINB7*-pEDD is the most prevalent hereditary PPK among East Asians due to a high prevalence of founder LOF variants in *SERPINB7* with an estimated prevalence of 1.2 per 10,000 in Japanese populations and 3.1 per 10,000 in Chinese populations (Huang et al. 2021). The nonsense variant c.796C > T (p.Arg266*) in *SERPINB7* is the most common founder LOF variant in Japanese and Chinese *SERPINB7*-pEDD patients (Kubo 2025). As to why a high carrier frequency of this LOF allele exists is not known, but it may have conferred some selective heterozygote advantage as postulated for other prevalent recessive gene variants (Man et al. 2007; Zlotogora. 2019; Xu et al. 2019).

Loss of *SERPINB7* inhibition may lead to the overactivation of its target protease legumain, a cysteine endopeptidase protease, impairing DSG1, DSC1, and keratin intermediate filaments (Li et al. 2025; Cohen-Barak et al. 2022). SERPINA12 is associated with inhibition of KLK7 activity via vaspin, leading to the decreased levels of KLK7 substrates, DSG1 and CDSN (Mohamad et al. 2020). Their common regulation of the desmosomal cadherin, DSG1, may provide a mechanistic link between these two serpins.

LOF pathogenic variants in *SERPINB8* are associated with autosomal recessive exfoliative ichthyosis (*SERPINB8*-pEDD, MIM #617115), also known as peeling skin syndrome 5 (PSS5), which is characterized by skin peeling, superficial scales, hyperkeratotic plaques, and erythema on the palms and soles (Pigors et al. 2016)*.* Its target substrate is furin, and study of cell culture models reveals a role in regulating desmosomal keratinocyte adhesion (Izaguirre et al. 2013; Pigors et al. 2016)*.*

LOF pathogenic variants in *SPINK5* (serine protease inhibitor of Kazal type 5) underlie the autosomal recessive skin condition termed Netherton syndrome [NS (*SPINK5*-sEDD), MIM #256500], which is clinically characterized by the triad of skin desquamation, atopic manifestation, and hair abnormalities (“bamboo-like” hair) (Fig. 1e; Netherton. 1958; Chavanas et al. 2000). Lympho-epithelial Kazal-type related inhibitor (LEKTI) is encoded by *SPINK5* (Bitoun et al. 2003). Loss of LEKTI leads to over-activation of skin KLKs (KLK5, KLK7, KLK14) and subsequently increased targeting of DSG1, DSC1, and CDSN for breakdown (Pontone et al. 2022). In *SPINK5*-sEDD, upregulated KLKs can activate the PAR-2 pathway, driving Th2-mediated skin inflammation skin, while barrier disruption facilitates pathogen penetration and triggers a Th17 immune response (Zani et al. 2022).

Autosomal recessive ichthyosis-hypotrichosis syndrome [(ARIH) *ST14*-sEDD, MIM #602400], which is characterized by generalized congenital scaling, diffuse non-scarring hypotrichosis, and photophobia, has been associated with sequence variants in *ST14* (type Ⅱ transmembrane serine protease matriptase) (Basel-Vanagaite et al. 2007; Paller et al. 2025). Matriptase deficiency leads to impaired degradation of corneodesmosomes within the stratum corneum, disrupted profilaggrin processing, abnormal hair follicle development, and increased apoptosis and marked depletion of thymocytes (List K et al. 2002; Alef et al. 2009). Matriptase has also been identified as an upstream activator of PAR-2, thereby triggering downstream signaling pathways that induce the expression of pro-inflammatory cytokines (Friis et al. 2017).

## Cysteine proteases and cystatins

Cathepsins belong to the lysosomal cysteine proteases of the papain-like family and are primarily responsible for intra-lysosomal protein degradation as well as mediating cellular housekeeping functions (Turk et al. 2001; Zeeuwen et al. 2009). They are often inhibited by cystatins (Abrahamson et al. 2003). Genetic studies of rare inherited monogenic skin disorders have also shown cysteine proteases and their inhibitors (cystatins) to have key functions in epidermal homeostasis and barrier function (Toomes et al. 1999; Ngcungcu et al. 2017).

For example, LOF variants in *CTSC* encoding Cathepsin C (CTSC) underlie two similar autosomal recessive skin syndromes termed Papillon-Lefèvre syndrome [PLS (*CTSC*-pEDD); MIM #245000] and Haim-Munk syndrome [HMS (*CTSC*-pEDD-arachnodactyly), MIM #245010], which both share clinical features of PPK and periodontal inflammation (Fig. 1f, g; Toomes et al. 1999; Ghanei et al. 2021; Hart et al. 2000; Chitsamankhun et al. 2024). Also, inherited genomic duplications upstream of the Cathepsin B gene (*CTSB*) are associated with keratolytic winter erythema [KWE (*CTSB*-pEDD), MIM #148370], characterized by palmoplantar erythema and epidermal peeling (Ngcungcu et al. 2017).

Loss of cathepsin inhibitor activity has also been associated with skin disorders/syndromes in which superficial peeling of the epidermis is the primary clinical phenotype. This indicates a role for this class of protease inhibitors in regulating epidermal adhesion. For example, LOF variants in the *CSTA* gene, encoding cystatin A, underlie an autosomal recessive form of acral peeling skin syndrome [APSS (*CSTA*-nEDD), MIM #607936], also called peeling skin syndrome 4 (PSS4), characterized by superficial palmoplantar exfoliation and erythroderma (Blaydon et al. 2011a). Cystatin A (also known as stefin A) has been shown to regulate cathepsin L, cathepsin V, and dust mite cysteine proteases (Der f1, Der p1) (Muttardi et al. 2016). In addition, cystatin M/E has been found to inhibit both lysosomal cysteine proteases, such as cathepsin L and cathepsin V, as well as the asparaginyl endopeptidase legumain, and is essential for maintaining epidermal differentiation as well as for the development of the hair follicles and the corneal epithelium in mice (Cheng et al. 2006; Zeeuwen et al. 2010). Pathogenetic variants in *CST6*, encoding cystatin M/E, underlie ectodermal dysplasia 15, hypohidrotic/hair type [ECTD15(*CST6*-sEDD), MIM #618535] and keratosis follicularis spinulosa decalvans [KFSD (*CST6*-sEDD-cicatricial), MIM #612843], both of which are characterized by generalized scaling, hypotrichosis, and photophobia (van den Bogaard et al. 2019; Eckl et al. 2021; Paller et al. 2025).

Calpains are calcium-dependent cysteine proteases that are highly expressed in stratified squamous epithelia and are inhibited by calpastatin, encoded by the *CAST* gene. LOF variants in *CAST* are associated with PLACK syndrome (*CAST*-pEDD, MIM #616295), which presents with peeling skin, leukonychia, acral punctate keratosis, cheilitis, and knuckle pads (Lin et al. 2015). In *CAST*-pEDD, the subsequent upregulated epidermal calpain activity seems to increase proteolysis of epidermal desmosomal components, induce keratinocyte apoptosis, and impair keratinocyte adhesion (Nguyen et al. 2024).

## Metalloproteinases

The metzincin superfamily of metalloproteinase primarily includes matrix metalloproteinases (MMPs), a disintegrin and metalloproteinases (ADAMs), ADAM with thrombospondin motifs (ADAMTS), and their endogenous inhibitors, known as tissue inhibitors of metalloproteases (TIMPs) (Rivera et al. 2019; Zhu 2021). These metalloproteinases play crucial roles in processing various extracellular matrix molecules and mediating cell signaling within the skin (Kümper et al. 2022).

ADAM17 (a disintegrin and metalloproteinase 17) is essential for processing numerous membrane-bound substrate proteins, including activating epidermal growth factor receptor (EGFR) ligands and releasing pro-inflammatory cytokines such as TNF-α (Rabinowitsch et al. 2023). Loss-of-function pathogenic variants in *ADAM17* are associated with inflammatory skin and bowel syndrome (*ADAM17*-sEDD, MIM #614328) (Blaydon et al. 2011b; Imoto et al. 2021; Chang et al. 2025). Gain-of-function variants in *RHBDF2* (encoding iRhom2 inactive rhomboid-like protein 2) underlie tylosis with oesophageal cancer [TOC (*RHBDF2*-pEDD), MIM #148500], characterized by PPK, oral and oesophageal leukoplakia, and a significantly high lifetime risk of oesophageal squamous cell carcinoma (Blaydon et al. 2012). Interestingly, ADAM17 protease activity, including EGFR signaling, is dependent on the “inactive” rhomboid protease iRhom2 (Adrain et al. 2012; Maretzky et al. 2013; Brooke et al. 2014).

MBTPS2 (membrane-bound transcription factor protease, site 2), also known as S2P (site-2 protease), is a membrane-embedded zinc metalloproteinase associated with lipogenesis and the endoplasmic reticulum stress response. S2P cleaves sterol regulatory element-binding proteins (SREBPs), which act as transcriptional factors involved in cholesterol metabolism (Haghighi et al. 2013). Variants in *MBTPS2* are linked to three X-linked recessive skin disorders: Olmsted syndrome [OS (*MBTPS2*-pEDD), MIM #300918], ichthyosis follicularis with atrichia and photophobia (IFAP) syndrome (with or without BRESHECK syndrome) (*MBTPS2*-sEDD-IFAP, MIM #308205), and keratosis follicularis spinulosa decalvans (KFSD (*MBTPS2*-sEDD-cicatricial alopecia), MIM #308800) (Haghighi et al. 2013; Oeffner et al. 2009; Naiki et al. 2012; Aten et al. 2010). These syndromes share the common clinical manifestation of keratotic skin, suggesting that pathogenic variants in *MBTPS2* reduce protease activity and impair sterol responsiveness in keratinocytes, thereby disrupting epidermal barrier function.

It should be noted that most cases of OS arise from pathogenic variants in the *TRPV3* (transient receptor potential vanilloid-3) gene, which can manifest in either autosomal dominant (gain-of-function) or recessive forms (Lin Z et al. 2012; Eytan et al. 2014).

## Concluding remarks

Proteases and their inhibitors are pivotal in skin barrier function, inflammation, and immune regulation. Dysregulated proteolytic activity contributes to a series of monogenic skin disorders with overlapping clinical and cellular phenotypes, often with impaired desmosomal function and inflammation. To date, the management of these disorders has remained largely supportive and symptomatic, underscoring the lack of disease-specific therapies. However, some clinical strategies have been tried in *SPINK5*-sEDD, including repurposing therapies linked to immune dysregulation, and used to treat more prevalent skin conditions such as psoriasis and atopic eczema. Monoclonal antibodies targeting IL-12/IL-23, IL-17, IL-4/IL-13, TNF-α, and IL1β have been trialed in *SPINK5*-sEDD (Samuelov et al. 2023; Gan et al. 2022; Blunder et al. 2025; Zingkou et al. 2022; Ragamin et al. 2023). In addition, JAK inhibitors have shown therapeutic benefit in some *SPINK5*-sEDD cases by dampening cytokine-mediated signaling and downstream inflammatory responses (Tang et al. 2025). Intravenous immunoglobulin has also demonstrated efficacy in *SPINK5*-sEDD through immunoglobulin replacement (Neema et al. 2023). Recently, GSK951, a KLK5 inhibitor, has been proposed as a promising therapeutic candidate (Liddle et al. 2021). Nevertheless, the current evidence for these therapeutic strategies remains largely limited to a small number of participants. Further multi-centre trials are warranted to establish the long-term efficacy of these therapies in *SPINK5*-sEDD.

Beyond recent advances in these targeted biologics and small-molecular immunomodulators, protease replacement strategies and protease inhibitor analogs represent emerging therapeutic avenues for conditions with this pathogenesis basis. Although steps have been made in elucidating protease-related mechanisms in genetic skin diseases, further research is needed for in-depth understanding of the disease pathogenesis and to facilitate the development of effective targeted treatments.

## Data Availability

No datasets were generated or analysed during the current study.

## References

[CR1] Abrahamson M, Alvarez-Fernandez M, Nathanson CM (2003) Cystatins. Biochem Soc Symp 70:179–199 10.1042/bss0700179 14587292

[CR2] Adrain C, Zettl M, Christova Y, Taylor N, Freeman M (2012) Tumor necrosis factor signaling requires iRhom2 to promote trafficking and activation of TACE. Science 335:225–228 22246777 10.1126/science.1214400 PMC3272371

[CR3] Akiyama M, Choate K, Hernández-Martín Á, Aldwin-Easton M, Bodemer C, Gostyński A, Hovnanian A, Ishida-Yamamoto A, Malovitski K, O’Toole EA, Paller AS, Schmuth M, Schwartz J, Sprecher E, Teng JMC, Granier Tournier C, Mazereeuw-Hautier J, Tadini G, Fischer J (2025) Nonsyndromic epidermal differentiation disorders: a new classification toward pathogenesis-based therapy. Br J Dermatol 193:619–641 40308026 10.1093/bjd/ljaf154

[CR4] Alef T, Torres S, Hausser I, Metze D, Türsen U, Lestringant GG, Hennies HC (2009) Ichthyosis, follicular atrophoderma, and hypotrichosis caused by mutations in ST14 is associated with impaired profilaggrin processing. J Invest Dermatol 129:862–869 18843291 10.1038/jid.2008.311

[CR5] Aten E, Brasz LC, Bornholdt D, Hooijkaas IB, Porteous ME, Sybert VP, Vermeer MH, Vossen RH, van der Wielen MJ, Bakker E, Breuning MH, Grzeschik KH, Oosterwijk JC, den Dunnen JT (2010) Keratosis follicularis spinulosa decalvans is caused by mutations in MBTPS2. Hum Mutat 31:1125–1133 20672378 10.1002/humu.21335

[CR6] Basel-Vanagaite L, Attia R, Ishida-Yamamoto A, Rainshtein L, Ben Amitai D, Lurie R, Pasmanik-Chor M, Indelman M, Zvulunov A, Saban S, Magal N, Sprecher E, Shohat M (2007) Autosomal recessive ichthyosis with hypotrichosis caused by a mutation in ST14, encoding type II transmembrane serine protease matriptase. Am J Hum Genet 80:467–477 17273967 10.1086/512487 PMC1821100

[CR7] Beck LA, Cork MJ, Amagai M, De Benedetto A, Kabashima K, Hamilton JD, Rossi AB (2002) Type 2 inflammation contributes to skin barrier dysfunction in atopic dermatitis. JID Innov 2:100131 10.1016/j.xjidi.2022.100131 PMC9428921 36059592

[CR8] Bitoun E, Micheloni A, Lamant L, Bonnart C, Tartaglia-Polcini A, Cobbold C, Al Saati T, Mariotti F, Mazereeuw-Hautier J, Boralevi F, Hohl D, Harper J, Bodemer C, D’Alessio M, Hovnanian A (2003) Lekti proteolytic processing in human primary keratinocytes, tissue distribution and defective expression in Netherton syndrome. Hum Mol Genet 12:2417–2430 12915442 10.1093/hmg/ddg247

[CR9] Blaydon DC, Nitoiu D, Eckl KM, Cabral RM, Bland P, Hausser I, van Heel DA, Rajpopat S, Fischer J, Oji V, Zvulunov A, Traupe H, Hennies HC, Kelsell DP (2011a) Mutations in CSTA, encoding Cystatin A, underlie exfoliative ichthyosis and reveal a role for this protease inhibitor in cell-cell adhesion. Am J Hum Genet 89:564–571 21944047 10.1016/j.ajhg.2011.09.001 PMC3188842

[CR10] Blaydon DC, Biancheri P, Di WL, Plagnol V, Cabral RM, Brooke MA, van Heel DA, Ruschendorf F, Toynbee M, Walne A, O’Toole EA, Martin JE, Lindley K, Vulliamy T, Abrams DJ, MacDonald TT, Harper JI, Kelsell DP (2011b) Inflammatory skin and bowel disease linked to ADAM17 deletion. N Engl J Med 365:1502–1508 22010916 10.1056/NEJMoa1100721

[CR11] Blaydon DC, Etheridge SL, Risk JM, Hennies HC, Gay LJ, Carroll R, Plagnol V, McRonald FE, Stevens HP, Spurr NK, Bishop DT, Ellis A, Jankowski J, Field JK, Leigh IM, South AP, Kelsell DP (2012) Rhbhf2 mutations are associated with tylosis, a familial esophageal cancer syndrome. Am J Hum Genet 90:340–346 22265016 10.1016/j.ajhg.2011.12.008 PMC3276661

[CR12] Blunder S, Hermann-Kleiter N, Mahmuti R, Hermann M, Ortner D, Reider D, Moosbrugger-Martinz V, Del Frari B, Stoitzner P, Dubrac S, Schmuth M, Gruber R (2025) Blocking of IL-4/IL-13 signalling with dupilumab results in restoration of serum and cutaneous abnormalities in Netherton Syndrome. Exp Dermatol 34:e70113 40344324 10.1111/exd.70113

[CR13] Brooke MA, Etheridge SL, Kaplan N, Simpson C, O’Toole EA, Ishida-Yamamoto A, Marches O, Getsios S, Kelsell DP (2014) *i*RHOM2-dependent regulation of ADAM17 in cutaneous disease and epidermal barrier function. Hum Mol Genet 23:4064–4076 24643277 10.1093/hmg/ddu120 PMC4110483

[CR14] Chang HR, Bannon M, Opper C, Slavotinek A, Hijazi G, Widmeyer KM, Kellner ES, Iqneibi M, Bayart C, Marathe KS, Bridges C (2025) Neonatal inflammatory skin and bowel disease 1 (NISBD1): a case of ADAM17 homozygous mutation. Pediatr Dermatol. 10.1111/pde.70028 40968583 10.1111/pde.70028

[CR15] Chavanas S, Bodemer C, Rochat A, Hamel-Teillac D, Ali M, Irvine AD, Bonafé JL, Wilkinson J, Taïeb A, Barrandon Y, Harper JI, de Prost Y, Hovnanian A (2000) Mutations in SPINK5, encoding a serine protease inhibitor, cause Netherton syndrome. Nat Genet 25:141–142 10835624 10.1038/75977

[CR16] Cheng T, Hitomi K, van Vlijmen-Willems IM, de Jongh GJ, Yamamoto K, Nishi K, Watts C, Reinheckel T, Schalkwijk J, Zeeuwen PL (2006) Cystatin M/E is a high affinity inhibitor of cathepsin V and cathepsin L by a reactive site that is distinct from the legumain-binding site. A novel clue for the role of cystatin M/E in epidermal cornification. J Biol Chem 281:15893–15899 16565075 10.1074/jbc.M600694200

[CR17] Chitsamankhun C, Siritongtaworn N, Fournier BPJ, Sriwattanapong K, Theerapanon T, Samaranayake L, Porntaveetus T (2024) Cathepsin C in health and disease: from structural insights to therapeutic prospects. J Transl Med 22:777 39164687 10.1186/s12967-024-05589-7 PMC11337848

[CR18] Cohen-Barak E, Godsel LM, Koetsier JL, Hegazy M, Kushnir-Grinbaum D, Hammad H, Danial-Farran N, Harmon R, Khayat M, Bochner R, Peled A, Rozenblat M, Krausz J, Sarig O, Johnson JL, Ziv M, Shalev SA, Sprecher E, Green KJ (2020) The role of desmoglein 1 in gap junction turnover revealed through the study of SAM Syndrome. J Invest Dermatol 140:556-567.e9 31465738 10.1016/j.jid.2019.08.433 PMC7039747

[CR19] Cohen-Barak E, Azzam W, Koetsier JL, Danial-Farran N, Barcan M, Hriesh M, Khayat M, Edison N, Krausz J, Gafni-Amsalem C, Kubo A, Godsel LM, Ziv M, Allon-Shalev S (2022) Acral peeling in Nagashima type palmo-plantar keratosis patients reveals the role of serine protease inhibitor B 7 in keratinocyte adhesion. Exp Dermatol 31:214–222 34379845 10.1111/exd.14444 PMC8831670

[CR20] Donzelli L, Bolgi O, Geiss-Friedlander R (2023) The amino-dipeptidyl peptidases DPP8 and DPP9: purification and enzymatic assays. Methods Enzymol 684:289–323 37230592 10.1016/bs.mie.2023.02.013

[CR21] Ebata A, Takeichi T, Nishida K, Chretien B, Miyazaki A, Yoshikawa T, Suzuki Y, Tanahashi K, Fukaura R, Seishima M, Suga Y, Muro Y, Nakazawa Y, Ogi T, Akiyama M (2025) Estimating the proportions of allele frequencies for SERPINA12 pathogenic variants in Japanese patients with Nagashima-type palmoplantar keratosis/keratoderma. Br J Dermatol 193:184–185 40138372 10.1093/bjd/ljaf111

[CR22] Eckl KM, Gruber R, Brennan L, Marriott A, Plank R, Moosbrugger-Martinz V, Blunder S, Schossig A, Altmüller J, Thiele H, Nürnberg P, Zschocke J, Hennies HC, Schmuth M (2021) Cystatin M/E variant causes autosomal dominant keratosis follicularis spinulosa decalvans by dysregulating cathepsins L and V. Front Genet 12:689940 34322157 10.3389/fgene.2021.689940 PMC8312243

[CR23] Eytan O, Fuchs-Telem D, Mevorach B, Indelman M, Bergman R, Sarig O, Goldberg I, Adir N, Sprecher E (2014) Olmsted syndrome caused by a homozygous recessive mutation in TRPV3. J Invest Dermatol 134:1752–1754 24463422 10.1038/jid.2014.37

[CR24] Fan J, Ning B, Lyon CJ, Hu TY (2017) Circulating peptidome and tumor-resident proteolysis. Enzymes 42:1–25 29054266 10.1016/bs.enz.2017.08.001

[CR25] Friis S, Tadeo D, Le-Gall SM, Jürgensen HJ, Sales KU, Camerer E, Bugge TH (2017) Matriptase zymogen supports epithelial development, homeostasis and regeneration. BMC Biol 15:46 28571576 10.1186/s12915-017-0384-4 PMC5452369

[CR26] Gan C, King E, Orchard D (2022) Secukinumab use in the treatment of Netherton’s syndrome. Australas J Dermatol 63:365–367 35622930 10.1111/ajd.13880

[CR27] Ghanei M, Abbaszadegan MR, Forghanifard MM, Aarabi A, Arab H (2021) A novel mutation in the cathepsin C (CTSC) gene in Iranian family with Papillon-Lefevre syndrome. Clin Exp Dent Res 7:568–573 33586345 10.1002/cre2.387 PMC8404484

[CR28] Haghighi A, Scott CA, Poon DS, Yaghoobi R, Saleh-Gohari N, Plagnol V, Kelsell DP (2013) A missense mutation in the MBTPS2 gene underlies the X-linked form of Olmsted syndrome. J Invest Dermatol 133:571–573 22931912 10.1038/jid.2012.289

[CR29] Hannula-Jouppi K, Harjama L, Einarsdottir E, Elomaa O, Kettunen K, Saarela J, Soronen M, Bouchard L, Lappalainen K, Heikkilä H, Kivirikko S, Seppänen MRJ, Kere J, Ranki A (2020) Nagashima-type palmoplantar keratosis in Finland caused by a SERPINB7 founder mutation. J Am Acad Dermatol 83:643–645 31706940 10.1016/j.jaad.2019.11.004

[CR30] Hart TC, Hart PS, Michalec MD, Zhang Y, Firatli E, Van Dyke TE, Stabholz A, Zlotogorski A, Shapira L, Soskolne WA (2000) Haim-Munk syndrome and Papillon-Lefèvre syndrome are allelic mutations in cathepsin C. J Med Genet 37:88–94 10662807 10.1136/jmg.37.2.88 PMC1734521

[CR31] Has C (2018) Peeling skin disorders: a paradigm for skin desquamation. J Invest Dermatol 138:1689–1691 30032785 10.1016/j.jid.2018.05.020

[CR32] Huang C, Yang Y, Huang X, Zhou Z (2021) Nagashima-type palmoplantar keratosis: clinical characteristics, genetic characterization, and clinical management. BioMed Res Int 2021:8841994 33575348 10.1155/2021/8841994 PMC7861918

[CR33] Hull PR, Hobbs A, Aron S, Ramsay M (2013) The elusive gene for keratolytic winter erythema. S Afr Med J 103:961–965 24300638 10.7196/samj.7253

[CR34] Imoto I, Saito M, Suga K, Kohmoto T, Otsu M, Horiuchi K, Nakayama H, Higashiyama S, Sugimoto M, Sasaki A, Homma Y, Shono M, Nakagawa R, Hayabuchi Y, Tange S, Kagami S, Masuda K (2021) Functionally confirmed compound heterozygous ADAM17 missense loss-of-function variants cause neonatal inflammatory skin and bowel disease 1. Sci Rep 11:9552 33953303 10.1038/s41598-021-89063-0 PMC8100128

[CR35] Irving JA, Pike RN, Dai W, Brömme D, Worrall DM, Silverman GA, Coetzer TH, Dennison C, Bottomley SP, Whisstock JC (2002) Evidence that serpin architecture intrinsically supports papain-like cysteine protease inhibition: engineering alpha(1)-antitrypsin to inhibit cathepsin proteases. Biochemistry 41:4998–5004 11939796 10.1021/bi0159985

[CR36] Izaguirre G, Qi L, Lima M, Olson ST (2013) Identification of serpin determinants of specificity and selectivity for furin inhibition through studies of α1PDX (α1-protease inhibitor Portland)-serpin B8 and furin active-site loop chimeras. J Biol Chem 288:21802–21814 23744066 10.1074/jbc.M113.462804 PMC3724637

[CR37] Jensen JM, Proksch E (2009) The skin’s barrier. G Ital Dermatol Venereol 144:689–700 19907407

[CR38] Jiang X, Yang C, Wang H, Cai L, Lin Z (2025) Nagashima-type palmoplantar keratosis patients harboring SERPINB7 and SERPINA12 variants. J Dermatol 52:e214–e215 39034590 10.1111/1346-8138.17399

[CR39] Kubo A (2025) History and prospects of Nagashima-type palmoplantar keratosis, the most common palmoplantar keratoderma in east Asian populations. J Dermatol 52:408–415 39749860 10.1111/1346-8138.17552 PMC11883851

[CR40] Kubo A, Shiohama A, Sasaki T, Nakabayashi K, Kawasaki H, Atsugi T, Sato S, Shimizu A, Mikami S, Tanizaki H, Uchiyama M, Maeda T, Ito T, Sakabe J, Heike T, Okuyama T, Kosaki R, Kosaki K, Kudoh J, Hata K, Umezawa A, Tokura Y, Ishiko A, Niizeki H, Kabashima K, Mitsuhashi Y, Amagai M (2013) Mutations in SERPINB7, encoding a member of the serine protease inhibitor superfamily, cause Nagashima-type palmoplantar keratosis. Am J Hum Genet 93:945–956 24207119 10.1016/j.ajhg.2013.09.015 PMC3824127

[CR41] Kümper M, Steinkamp J, Zigrino P (2022) Metalloproteinases in dermal homeostasis. Am J Physiol Cell Physiol 323:C1290–C1303 36094433 10.1152/ajpcell.00450.2021

[CR42] Li Z, Xie S, Xu X, Chen Z, Wang L, Yang Y, Wang S (2025) Phenotypic and genotypic analysis of SERPINA12-related autosomal recessive palmoplantar keratoderma in southwestern China. J Dermatol 52:545–550 39663865 10.1111/1346-8138.17581

[CR43] Liddle J, Beneton V, Benson M, Bingham R, Bouillot A, Boullay AB, Brook E, Cryan J, Denis A, Edgar E, Ferrie A, Fouchet MH, Grillot D, Holmes DS, Howes A, Krysa G, Laroze A, Lennon M, McClure F, Moquette A, Nicodeme E, Santiago B, Santos L, Smith KJ, Thorpe JH, Thripp G, Trottet L, Walker AL, Ward SA, Wang Y, Wilson S, Pearce AC, Hovnanian A (2021) A potent and selective kallikrein-5 inhibitor delivers high pharmacological activity in skin from patients with Netherton syndrome. J Invest Dermatol 141:2272–2279 33744298 10.1016/j.jid.2021.01.029

[CR44] Lin Z, Chen Q, Lee M, Cao X, Zhang J, Ma D, Chen L, Hu X, Wang H, Wang X, Zhang P, Liu X, Guan L, Tang Y, Yang H, Tu P, Bu D, Zhu X, Wang K, Li R, Yang Y (2012) Exome sequencing reveals mutations in TRPV3 as a cause of Olmsted syndrome. Am J Hum Genet 90:558–564 22405088 10.1016/j.ajhg.2012.02.006 PMC3309189

[CR45] Lin Z, Zhao J, Nitoiu D, Scott CA, Plagnol V, Smith FJ, Wilson NJ, Cole C, Schwartz ME, McLean WH, Wang H, Feng C, Duo L, Zhou EY, Ren Y, Dai L, Chen Y, Zhang J, Xu X, O’Toole EA, Kelsell DP, Yang Y (2015) Loss-of-function mutations in CAST cause peeling skin, leukonychia, acral punctate keratoses, cheilitis, and knuckle pads. Am J Hum Genet 96:440–447 25683118 10.1016/j.ajhg.2014.12.026 PMC4375526

[CR46] List K, Haudenschild CC, Szabo R, Chen W, Wahl SM, Swaim W, Engelholm LH, Behrendt N, Bugge TH (2002) Matriptase/MT-SP1 is required for postnatal survival, epidermal barrier function, hair follicle development, and thymic homeostasis. Oncogene 21:3765–3779 12032844 10.1038/sj.onc.1205502

[CR47] Liu J, Chen Z, Hu L, Song Z, Mo R, Tsang LS, Liu Y, Huang X, Gong Z, Lin Z, Yang Y (2023) Investigation of Nagashima-type palmoplantar keratoderma in China: a cross-sectional study of 234 patients. J Dermatol 50:375–382 36317385 10.1111/1346-8138.16621

[CR48] Liu Y, Liu J, Chen Y, Mo R, Xiang R, Song Z, Yang Y, Chen Z (2024) Potential digenic inheritance of SERPINB7 and SERPINA12 variants in Chinese patients with Nagashima-type palmoplantar keratosis. Br J Dermatol 191:136–138 38529670 10.1093/bjd/ljae134

[CR49] Man YK, Trolove C, Tattersall D, Thomas AC, Papakonstantinopoulou A, Patel D, Scott C, Chong J, Jagger DJ, O’Toole EA, Navsaria H, Curtis MA, Kelsell DP (2007) A deafness-associated mutant human connexin 26 improves the epithelial barrier in vitro. J Membr Biol 218:29–37 17581693 10.1007/s00232-007-9025-0 PMC2845879

[CR50] Maretzky T, McIlwain DR, Issuree PD, Li X, Malapeira J, Amin S, Lang PA, Mak TW, Blobel CP (2013) iRhom2 controls the substrate selectivity of stimulated ADAM17-dependent ectodomain shedding. Proc Natl Acad Sci U S A 110:11433–11438 23801765 10.1073/pnas.1302553110 PMC3710827

[CR51] Meyer-Hoffert U (2009) Reddish, scaly, and itchy: how proteases and their inhibitors contribute to inflammatory skin diseases. Arch Immunol Ther Exp (Warsz) 57:345–354 19688185 10.1007/s00005-009-0045-6

[CR52] Mohamad J, Sarig O, Malki L, Rabinowitz T, Assaf S, Malovitski K, Shkury E, Mayer T, Vodo D, Peled A, Daniely D, Pavlovsky M, Shomron N, Samuelov L, Sprecher E (2020) Loss-of-function variants in SERPINA12 underlie autosomal recessive palmoplantar keratoderma. J Invest Dermatol 140:2178–2187 32247861 10.1016/j.jid.2020.02.030

[CR53] Muttardi K, Nitoiu D, Kelsell DP, O’Toole EA, Batta K (2016) Acral peeling skin syndrome associated with a novel CSTA gene mutation. Clin Exp Dermatol 41:394–398 26684698 10.1111/ced.12777

[CR54] Naiki M, Mizuno S, Yamada K, Yamada Y, Kimura R, Oshiro M, Okamoto N, Makita Y, Seishima M, Wakamatsu N (2012) MBTPS2 mutation causes BRESEK/BRESHECK syndrome. Am J Med Genet A 158A:97–102 22105905 10.1002/ajmg.a.34373

[CR55] Neema S, Vasudevan B, Rathod A, Mukherjee S, Vendhan S, Gera V (2023) Intravenous immunoglobulin for the management of Netherton syndrome. Indian J Dermatol Venereol Leprol 89:754–756 37317770 10.25259/IJDVL_558_2022

[CR56] Netherton EW (1958) A unique case of trichorrhexis nodosa: bamboo hairs. Arch Dermatol 78:483–487 10.1001/archderm.1958.01560100059009 13582191

[CR57] Ngcungcu T, Oti M, Sitek JC, Haukanes BI, Linghu B, Bruccoleri R, Stokowy T, Oakeley EJ, Yang F, Zhu J, Sultan M, Schalkwijk J, van Vlijmen-Willems IMJJ, von der Lippe C, Brunner HG, Ersland KM, Grayson W, Buechmann-Moller S, Sundnes O, Nirmala N, Morgan TM, van Bokhoven H, Steen VM, Hull PR, Szustakowski J, Staedtler F, Zhou H, Fiskerstrand T, Ramsay M (2017) Duplicated enhancer region increases expression of CTSB and segregates with keratolytic winter erythema in south African and Norwegian families. Am J Hum Genet 100:737–750 28457472 10.1016/j.ajhg.2017.03.012 PMC5420352

[CR58] Nguyen C, Hughes C, Little S, Carruth A, Nolan D, Ruth J (2024) CASTing the net wider: a case report of PLACK syndrome associated with dilated cardiomyopathy. Pediatr Dermatol 41:1211–1214 38994911 10.1111/pde.15671

[CR59] Oeffner F, Fischer G, Happle R, König A, Betz RC, Bornholdt D, Neidel U, Boente MC, Redler S, Romero-Gomez J, Salhi A, Vera-Casaño A, Weirich C, Grzeschik KH (2009) IFAP syndrome is caused by deficiency in MBTPS2, an intramembrane zinc metalloprotease essential for cholesterol homeostasis and ER stress response. Am J Hum Genet 84:459–467 19361614 10.1016/j.ajhg.2009.03.014 PMC2667992

[CR60] Paller AS, Teng J, Mazereeuw-Hautier J, Hernández-Martín Á, Granier Tournier C, Hovnanian A, Aldwin-Easton M, Tadini G, Schwartz J, Sprecher E, Malovitski K, Ishida-Yamamoto A, Choate K, Akiyama M, O’Toole EA, Fischer J, Bodemer C, Gostynski A, Schmuth M (2025) Syndromic epidermal differentiation disorders: a new classification toward pathogenesis-based therapy. Br J Dermatol 193:592–618 40184496 10.1093/bjd/ljaf123

[CR61] Pigors M, Sarig O, Heinz L, Plagnol V, Fischer J, Mohamad J, Malchin N, Rajpopat S, Kharfi M, Lestringant GG, Sprecher E, Kelsell DP, Blaydon DC (2016) Loss-of-function mutations in SERPINB8 linked to exfoliative ichthyosis with impaired mechanical stability of intercellular adhesions. Am J Hum Genet 99:430–436 27476651 10.1016/j.ajhg.2016.06.004 PMC4974070

[CR62] Pontone M, Giovannini M, Filippeschi C, Oranges T, Pedaci FA, Mori F, Barni S, Barbati F, Consonni F, Indolfi G, Lodi L, Azzari C, Ricci S, Hovnanian A (2022) Biological treatments for pediatric Netherton syndrome. Front Pediatr 10:1074243 36619513 10.3389/fped.2022.1074243 PMC9822572

[CR63] Rabinowitsch AI, Maretzky T, Weskamp G, Haxaire C, Tueshaus J, Lichtenthaler SF, Monette S, Blobel CP (2023) Analysis of the function of ADAM17 in iRhom2 curly-bare and tylosis with esophageal cancer mutant mice. J Cell Sci 136:jcs260910 37282854 10.1242/jcs.260910 PMC10357010

[CR64] Ragamin A, Nouwen AEM, Dalm VASH, van Mierlo MMF, Lincke CR, Pasmans SGMA (2023) Treatment experiences with intravenous immunoglobulins, ixekizumab, dupilumab, and anakinra in Netherton syndrome: a case series. Dermatology 239:72–80 35998563 10.1159/000525987 PMC9909717

[CR65] Ray CA, Black RA, Kronheim SR, Greenstreet TA, Sleath PR, Salvesen GS, Pickup DJ (1992) Viral inhibition of inflammation: cowpox virus encodes an inhibitor of the interleukin-1 beta converting enzyme. Cell 69:597–604 1339309 10.1016/0092-8674(92)90223-y

[CR66] Rivera S, García-González L, Khrestchatisky M, Baranger K (2019) Metalloproteinases and their tissue inhibitors in Alzheimer’s disease and other neurodegenerative disorders. Cell Mol Life Sci 76:3167–3191 31197405 10.1007/s00018-019-03178-2 PMC11105182

[CR67] Samuelov L, Shehadeh W, Sarig O, Gat A, Matz H, Sprecher E (2023) Ustekinumab therapy for Netherton syndrome. J Dermatol 50:494–499 36419401 10.1111/1346-8138.16645

[CR68] Sil BK, Jamiruddin MR, Paul PK, Aekwattanaphol N, Nakpheng T, Haq MA, Buatong W, Srichana T (2024) Ascorbic acid as serine protease inhibitor in lung cancer cell line and human serum albumin. PLoS ONE 19:e0303706 39042609 10.1371/journal.pone.0303706 PMC11265676

[CR69] Sprecher E, Ishida-Yamamoto A, Schwartz J, Akiyama M, Aldwin-Easton M, Choate K, Fischer J, Gostyński A, Granier Tournier C, Hernández-Martín Á, Hovnanian A, Malovitski K, Mazereeuw-Hautier J, Paller AS, Schmuth M, Tadini G, Teng J, Bodemer C, O’Toole EA (2025) Palmoplantar epidermal differentiation disorders: a new classification toward pathogenesis-based therapy. Br J Dermatol 193:364–380 40106577 10.1093/bjd/ljaf054

[CR70] Stamatas GN (2024) Protein degradation in the stratum corneum. Int J Cosmet Sci 46:590–597 39113293 10.1111/ics.12974

[CR71] Stewart-McGuinness C, Platt CI, Ozols M, Goh B, Griffiths TW, Sherratt MJ (2022) Defining the protease and protease inhibitor (P/PI) proteomes of healthy and diseased human skin by modified systematic review. Biomolecules 12:475 35327667 10.3390/biom12030475 PMC8946613

[CR72] Tang JT, Qin YL, Zhao WJ, Tu Y, Sun DJ (2025) Abrocitinib alleviates the symptoms of Netherton syndrome and is well tolerated. J Dermatol Treat 36:2447883 10.1080/09546634.2024.2447883 40159127

[CR73] Toomes C, James J, Wood AJ, Wu CL, McCormick D, Lench N, Hewitt C, Moynihan L, Roberts E, Woods CG, Markham A, Wong M, Widmer R, Ghaffar KA, Pemberton M, Hussein IR, Temtamy SA, Davies R, Read AP, Sloan P, Dixon MJ, Thakker NS (1999) Loss-of-function mutations in the cathepsin C gene result in periodontal disease and palmoplantar keratosis. Nat Genet 23:421–424 10581027 10.1038/70525

[CR74] Turk V, Turk B, Turk D (2001) Lysosomal cysteine proteases: facts and opportunities. EMBO J 20:4629–4633 11532926 10.1093/emboj/20.17.4629 PMC125585

[CR75] Ulbricht D, Tindall CA, Oertwig K, Hanke S, Sträter N, Heiker JT (2018) Kallikrein-related peptidase 14 is the second KLK protease targeted by the serpin vaspin. Biol Chem 399:1079–1084 29494334 10.1515/hsz-2018-0108

[CR76] van den Bogaard EHJ, van Geel M, van Vlijmen-Willems IMJJ, Jansen PAM, Peppelman M, van Erp PEJ, Atalay S, Venselaar H, Simon MEH, Joosten M, Schalkwijk J, Zeeuwen PLJM (2019) Deficiency of the human cysteine protease inhibitor cystatin M/E causes hypotrichosis and dry skin. Genet Med 21:1559–1567 30425301 10.1038/s41436-018-0355-3 PMC6752276

[CR77] Xu K, Kosoy R, Shameer K, Kumar S, Liu L, Readhead B, Belbin GM, Lee HC, Chen R, Dudley JT (2019) Genome-wide analysis indicates association between heterozygote advantage and healthy aging in humans. BMC Genet 20:52 31266448 10.1186/s12863-019-0758-4 PMC6604157

[CR78] Zani MB, Sant’Ana AM, Tognato RC, Chagas JR, Puzer L (2022) Human tissue kallikreins-related peptidases are targets for the treatment of skin desquamation diseases. Front Med (Lausanne) 8:777619 35356049 10.3389/fmed.2021.777619 PMC8959125

[CR79] Zeeuwen PL, Cheng T, Schalkwijk J (2009) The biology of cystatin M/E and its cognate target proteases. J Invest Dermatol 129:1327–1338 19262604 10.1038/jid.2009.40

[CR80] Zeeuwen PL, van Vlijmen-Willems IM, Cheng T, Rodijk-Olthuis D, Hitomi K, Hara-Nishimura I, John S, Smyth N, Reinheckel T, Hendriks WJ, Schalkwijk J (2010) The cystatin M/E-cathepsin L balance is essential for tissue homeostasis in epidermis, hair follicles, and cornea. FASEB J 24:3744–3755 20495178 10.1096/fj.10-155879

[CR81] Zhang C, Wang Y, Cao Q, Ge H, Lin X, Wang X, Zhao A, He W, Zeng Q, Huang H, Yan Q, Li M (2025) Serpina12 variants harboured in Nagashima-type palmoplantar keratoderma: potential inflammatory characteristics. J Dermatol Sci 118:104–106 40312186 10.1016/j.jdermsci.2025.04.009

[CR82] Zhu Y (2021) Metalloproteases in gonad formation and ovulation. Gen Comp Endocrinol 314:113924 34606745 10.1016/j.ygcen.2021.113924 PMC8576836

[CR83] Zingkou E, Pampalakis G, Sotiropoulou G (2022) Cocktails of KLK5 protease inhibitors and anti-TNFα therapeutics: an effective treatment for Netherton syndrome. J Clin Immunol 42:597–605 35040012 10.1007/s10875-021-01195-0

[CR84] Zlotogora J (2019) Autosomal recessive diseases among the Israeli Arabs. Hum Genet 138:1117–1122 31243543 10.1007/s00439-019-02043-3

